# Modified single-incision MIS-TLIF with expandable tubular assistance for degenerative lumbar spine diseases

**DOI:** 10.3389/fsurg.2024.1482067

**Published:** 2025-01-06

**Authors:** Wenlong Hu, Fei He, Kai Sun, Haiwu Wan, Sijun Ruan, Bo Huang

**Affiliations:** ^1^ Department of Orthopedic Surgery, Jiujiang University Affiliated Hospital, Jiujiang, China; ^2^ Jiujiang Orthopedic Medical Quality Control Center, Jiangxi, China

**Keywords:** degenerative lumbar spine disease, minimally invasive surgery, lumbar interbody fusion, single-incision MIS-TLIF, expandable tubular assistance

## Abstract

**Objective:**

Evaluating the clinical value of the modified single-incision posterior median approach with expandable tubular assistance for lumbar interbody fusion in managing degenerative lumbar spine diseases.

**Method:**

A retrospective analysis was conducted on 121 patients with single-level degenerative lumbar spine disease treated in our spine surgery department from January 2017 to December 2021. Of these, 72 patients underwent a modified single-incision posterior median approach with expandable tubular assistance lumbar interbody fusion (single-incision MIS-TLIF group), while 49 patients received the classic open posterior median incision P-TLIF (open surgery group). We collected basic demographic data including age, gender, BMI, and surgical level. Surgical-related indicators such as operation time, intraoperative blood loss, postoperative drainage, length of hospital stay, hospital costs, and complication rates were compared between the two groups. Laboratory results [whole blood C-reactive protein (CRP), serum creatine kinase (CK)] and clinical outcomes [VAS scores for low back and leg pain, Oswestry Disability Index (ODI), excellent and good rate according to the modified MacNab criteria, and interbody fusion rate according to the Brantigan criteria] were also evaluated.

**Result:**

There were no significant differences in the basic demographics between the two groups. The operation time, postoperative hospital stay, and hospital costs were also similar between the groups. However, significant differences were observed in intraoperative blood loss, postoperative drainage, and complication rates. On postoperative days 1 and 3, whole blood CRP and CK levels showed marked differences between the groups. At 3, 6, and 12 months postoperatively, the single-incision MIS-TLIF group had lower ODI scores and VAS scores for back pain compared to the open surgery group. The excellent and good rate according to the MacNab criteria was higher in the single-incision MIS-TLIF group. There were no significant differences in leg pain VAS scores and interbody fusion rates at 12 months postoperatively between the groups.

**Conclusion:**

The modified single-incision posterior median approach with expandable tubular assistance lumbar interbody fusion is highly effective in treating degenerative lumbar spine diseases. It results in less postoperative pain, faster recovery, and significant improvement in postoperative functional outcomes, making it a valuable treatment option.

## 1 Introduction

Globally, the aging population has led to an increase in degenerative lumbar spine diseases(DLSS) (1, 2). Research indicates that the prevalence of these conditions in the elderly population may reach 103 million, severely impacting health-related quality of life (HR-QoL) (3). Studies show that the incidence of degenerative lumbar spine diseases even exceeds that of osteoarthritis in the knee and hip joints, cardiovascular diseases, cerebrovascular diseases, and respiratory diseases (4, 5). Degenerative changes in the lumbar spine are believed to result from disc dehydration, bulging, and space collapse, leading to narrowed intervertebral spaces (6). This increases stress transfer to the facet joints, accelerating cartilage degeneration and osteophyte formation, ultimately causing spinal canal stenosis (7, 8). Additionally, degenerative changes in the discs and facet joints can lead to central canal or lateral recess stenosis, vertebral displacement, and degenerative spondylolisthesis, which may compress nerves and manifest clinically as lower back and leg pain, numbness, weakness, and gait disturbances (9–11).

Degenerative lumbar spine diseases predominantly affect middle-aged and elderly populations, with conservative treatment being the primary approach. However, severe cases may require surgical intervention, and the main treatment for lumbar spinal stenosis is surgery, with various surgical methods available (12). Traditional fusion techniques include Posterior Lumbar Interbody Fusion (PLIF), Transforaminal Lumbar Interbody Fusion (TLIF), Anterior Lumbar Interbody Fusion (ALIF), Oblique Lateral Lumbar Interbody Fusion (OLIF), and endoscopic lumbar interbody fusion (13). With advancements in spine surgery, procedures are increasingly becoming minimally invasive. Recently developed minimally invasive spinal fusion techniques include OLIF, MIS-TLIF, and endoscopic lumbar interbody fusion (14).

Studies have reported that minimally invasive posterior lumbar interbody fusion offers advantages such as less trauma, faster recovery, and higher fusion rates (15). It allows direct decompression and fusion under direct vision with the assistance of a unilateral facet joint resection. However, classical MIS-TLIF often requires x-ray monitoring and percutaneous pedicle screw fixation, which is very expensive in China, limiting its development in economically underdeveloped areas.

In this study, the authors have made modifications to the procedure, completing the MIS-TLIF through a single posterior median incision and using conventional pedicle screws for fixation. This retrospective analysis compares data from patients with degenerative lumbar spine diseases (DLSS) treated with single-incision posterior median approach lumbar interbody fusion and traditional open surgery, evaluating the efficacy and safety of these two surgical methods to provide a reference for the clinical surgical treatment of DLSS.

## 2 Method

This retrospective study was approved by the Ethics Committee of the Jiujiang university affiliated hospital. The study included 121 patients who underwent single segment lumbar fusion surgery between January 2017 and December 2021.The choice of surgical method was largely based on the surgeon's preference and the availability of expandable channels, randomness exists throughout the process(Ethics approval number:NO.jjumer-a-2024–0105).All patients provided written informed consent.Inclusion criteria were: (1) diagnosis of single segment lumbar spinal stenosis, lumbar disc protrusion, or Grade I lumbar spondylolisthesis; (2) surgical approach of either posterior open P-TLIF or single-incision MIS-TLIF; (3) complete follow-up for 12 months. Exclusion criteria were: (1) history of previous spinal surgery; (2) diseases affecting bone metabolism, such as chronic renal failure or hyperparathyroidism; (3) other spinal or spinal cord diseases, such as spinal cord injury, epidural hematoma or abscess, or metastatic diseases; (4) incomplete follow-up data, poor patient compliance, and low cooperation.

## 3 Surgical process

All patients underwent general anesthesia administered by the same team of surgeons. Once anesthesia was effective, the patients were positioned correctly in the prone position, and the surgical segment was localized under x-ray guidance.

### 3.1 Open surgery

A longitudinal incision approximately 8 cm long was made along the posterior midline. The tissues were separated layer by layer, and the paraspinal muscles on both sides were stripped to expose the facet joints and lamina. Following anatomical landmarks, punctures were made, and guide pins were inserted. After confirming the proper position of the guide pins under fluoroscopy, pedicle screws of the appropriate length were implanted. The inferior articular process, part of the superior articular process, and part of the lamina above and below were removed, and bone fragments were collected. The intervertebral foramen and lateral recess were exposed to reveal the compressed nerve root.

**Figure 1:**
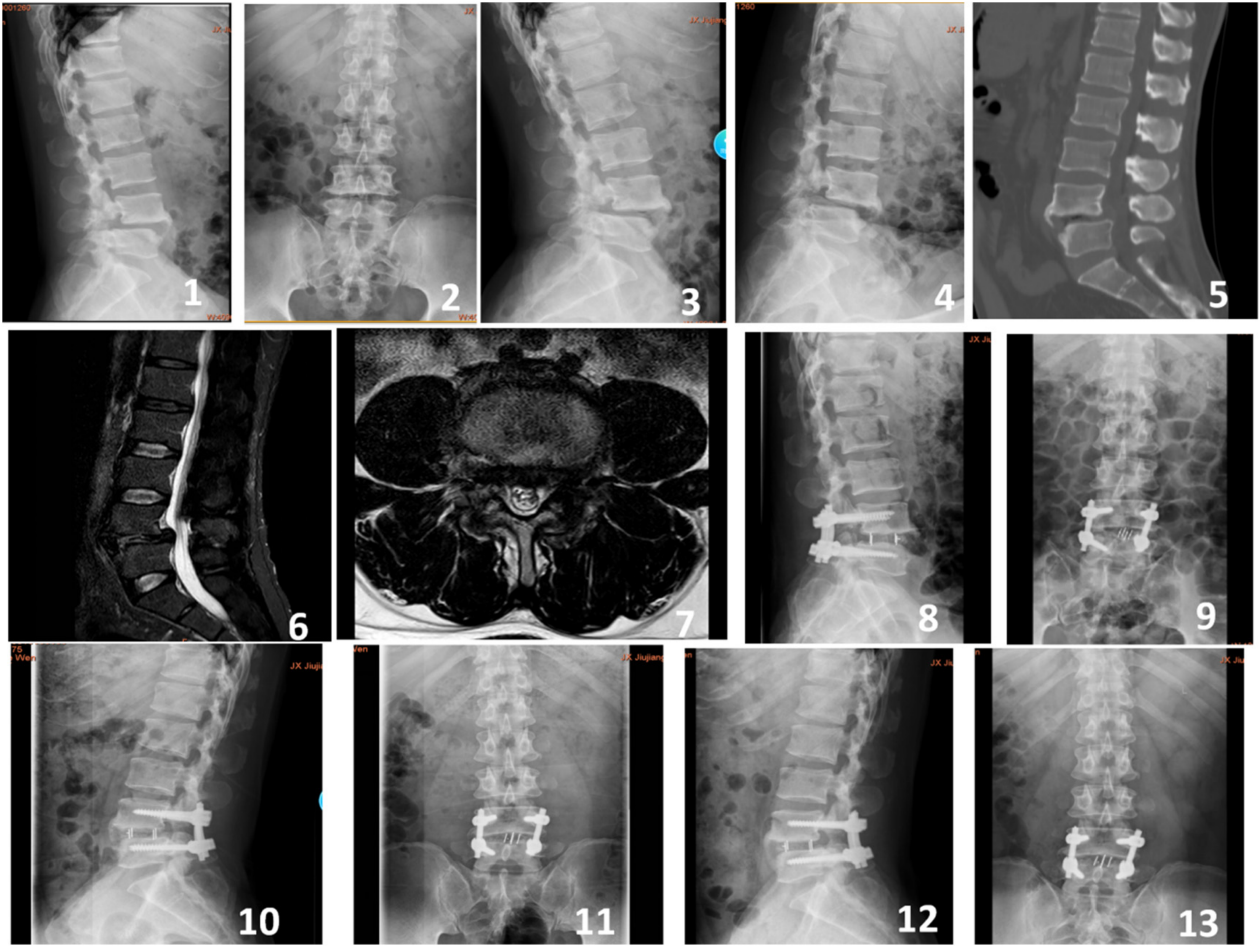
A 55-year-old male presented with recurrent lower back pain accompanied by intermittent claudication for 4 years. Preoperative imaging included lateral, anteroposterior, and dynamic flexion-extension lumbar radiographs (Figures 1-4), preoperative CT scan (Figure 5), and preoperative MRI (Figures 6-7). Postoperative imaging at 3 days following the open surgery included lateral and anteroposterior lumbar radiographs (Figures 8-9). Follow-up imaging at 3 months (Figures 10-11) and 12 months (Figures 12-13) postoperatively showed good bony fusion on lateral and anteroposterior lumbar radiographs.

The nerve root was protected while intervertebral distractors were placed sequentially. The annulus fibrosus, nucleus pulposus, and cartilage endplates were adequately removed and cleaned from the intervertebral space. Autologous bone and a suitable PEEK interbody fusion device were implanted. The nerve root was re-examined, and the size and position of the interbody fusion device were confirmed under fluoroscopy. The pedicle screws were connected with rods and fixed in place. The surgical area was irrigated, a drainage tube was placed, and the incision was closed in layers (Figure 1).

### 3.2 Single-Incision MIS-TLlF

**Figure 2:**
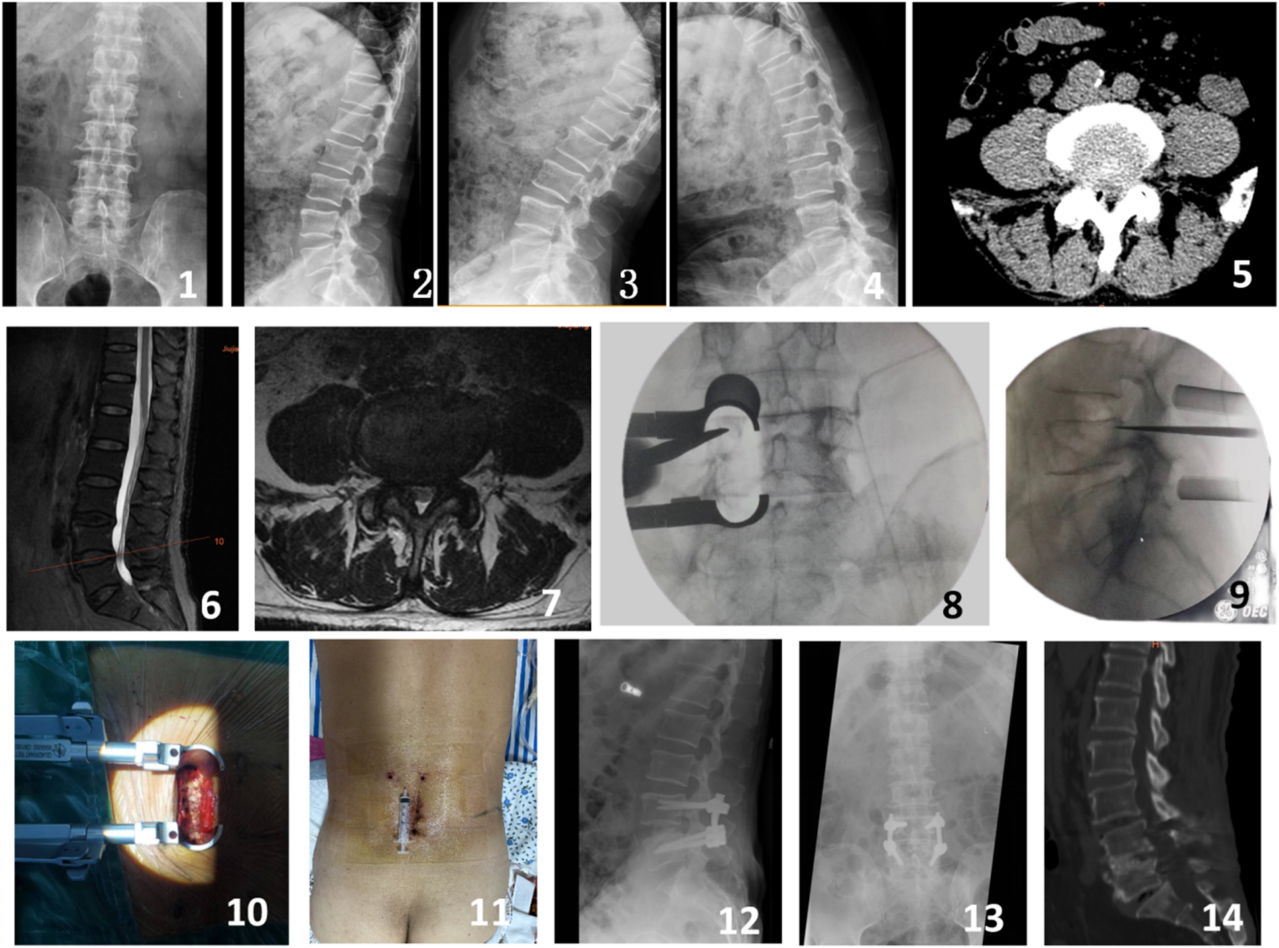
A 68-year-old male presented with intermittent claudication and numbness in the left lower limb for 4 years. Preoperative imaging included lateral, anteroposterior, and dynamic flexion-extension lumbar radiographs (Figures 1-4), preoperative CT scan (Figure 5), and preoperative MRI (Figures 6-7). Intraoperative images captured the process of the modified single-incision surgery (Figures 8-10). The postoperative incision was sutured and measured approximately 5 cm (Figure 11). Postoperative imaging at 3 days following surgery included lateral and anteroposterior lumbar radiographs (Figures 12-13). A CT scan at 12 months postoperatively showed good bony fusion (Figure 14).

**Figure 3:**
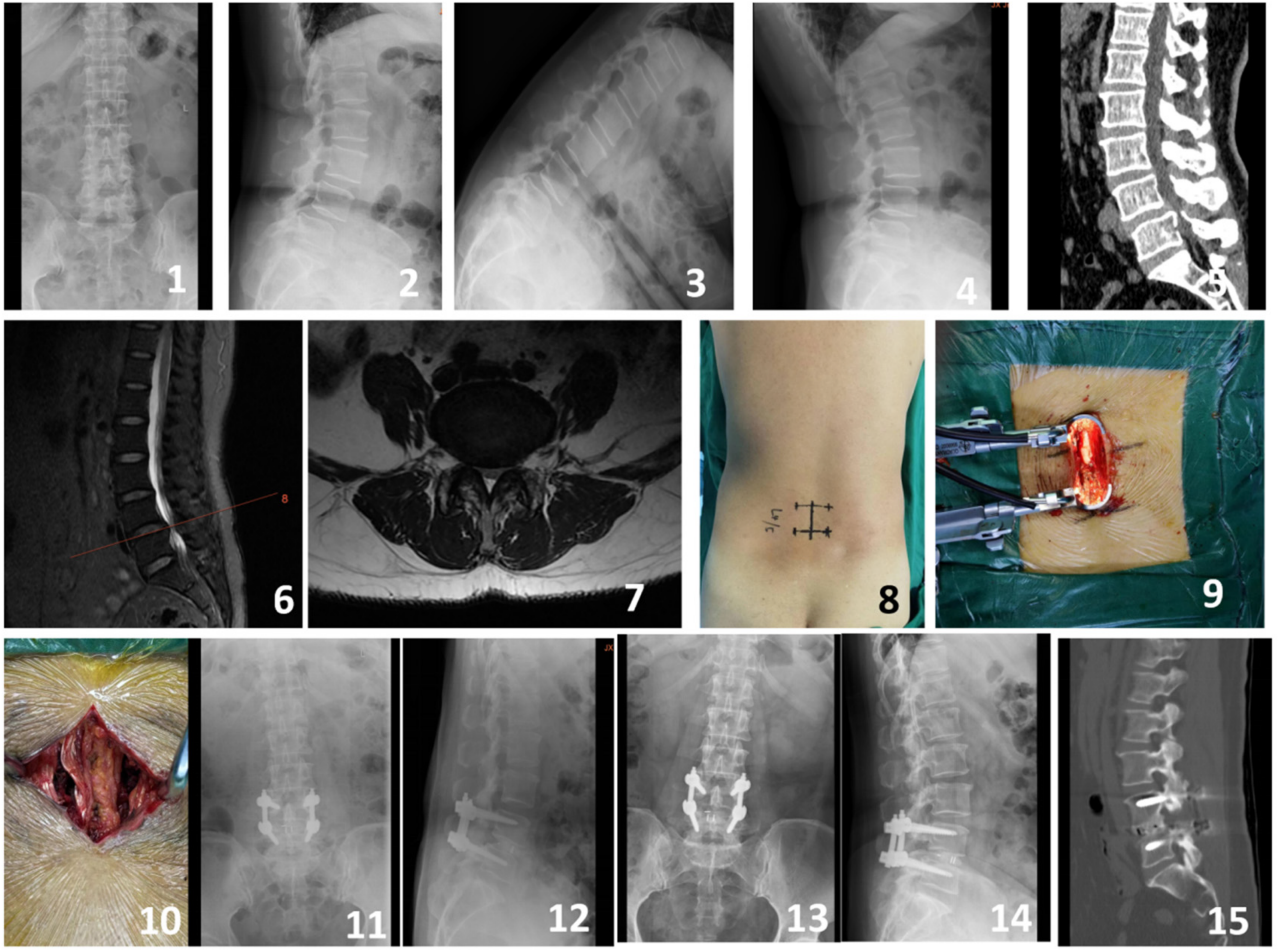
A 64-year-old male presented with intermittent claudication and numbness in the right lower limb for 2 years. Preoperative imaging included lateral, anteroposterior, and dynamic flexion-extension lumbar radiographs (Figures 1-4), preoperative CT scan (Figure 5), and preoperative MRI (Figures 6-7). Intraoperative images captured the process of the modified single-incision surgery (Figures 8-10). Postoperative imaging at 3 days following surgery included lateral and anteroposterior lumbar radiographs (Figures 11-12). Follow-up imaging at 6 months postoperatively included lateral and anteroposterior lumbar radiographs (Figures 13-14). A CT scan at 12 months postoperatively showed good bony fusion (Figure 15).

A midline incision was made with the intervertebral space extending 2 cm above and below. The skin, subcutaneous tissue, and lumbodorsal fascia were incised, and the muscle gaps were separated. A expandable tubular (Medtronic Quadrant) was inserted into the gap and fixed with with an attached light source and lens,exposing the target lumbar lamina and facet joints. Punctures were made, and guide pins were inserted. Once the guide pin position was confirmed under fluoroscopy, the pins were removed, and bone wax was used to seal the pinholes. The facet joints and lamina were removed under the channel, exposing the affected intervertebral space. The intervertebral space was treated similarly to the open surgery. The excised lamina bone fragments were placed into the oblique opening of the channel and gently hammered into place between the upper and lower vertebral bodies. The PEEK fusion device was gently tapped into the intervertebral space containing the bone fragments. The position of the fusion device was confirmed under fluoroscopy with a C-arm x-ray machine.Pedicle screws were implanted through the pre-drilled guide pinholes. After separating the muscle gap on the opposite side, guide pins were directly implanted, and their position was confirmed under fluoroscopy. Pedicle screws were then inserted. The wound was irrigated, and the surgical incision was closed in layers. A negative pressure drainage tube was placed below the surgical field for drainage (Figures 2,3).

### 3.3 Perioperative management

Both groups received antibiotics 30 min before surgery and for 48 h postoperatively to prevent infection. Postoperatively, short-term use of nonsteroidal anti-inflammatory drugs (NSAIDs) was administered for pain relief. Based on the Autar DVT risk assessment scale, low molecular weight heparin was administered as needed. Postoperative management included monitoring drainage volume, with the drainage tube being removed when the output was less than 30 ml. Patients were encouraged to begin early ambulation with the protection of a brace. Early rehabilitation training was conducted under the guidance of professional rehabilitation physicians for both groups.

### 3.4 Assessment criteria

Compare the surgical duration, intraoperative blood loss, postoperative drainage, postoperative length of stay, hospitalization costs, and complication rates between the two groups of patients. Compare the preoperative, postoperative 1 day, and postoperative 3 days CRP and serum CK levels in both groups of patients to evaluate the degree of surgical muscle injury. Collect and compare the preoperative, postoperative 3 months, 6 months, and 12 months VAS scores for low back and leg pain in both groups to evaluate the efficacy, and the last follow-up was performed using the modified MacNab standard to classify the efficacy as excellent, good, fair, or poor. The modified Brantigan scoring system was used to evaluate the intervertebral fusion, and the postoperative 6-month and 12-month x-ray images were used to evaluate the intervertebral fusion. The Brantigan scoring criteria were as follows: 4 points, complete fusion, good contour, and the appearance of continuous callus; 3 points, good fusion, but there was still a faint translucent line; 2 points, continuous callus in the upper and lower parts (50%), but there was still a lot of translucent line; 1 point, the upper and lower parts were not connected, but the bone volume was more than the postoperative intervertebral bone graft volume; 0 points, the intervertebral bone graft was absorbed, the intervertebral space height decreased, and the vertebral body did not fuse. Modified Brantigan scoring ≥3 points was considered intervertebral fusion.

### 3.5 Statistical approach

Statistical analysis was performed using SPSS 24.0 software. Continuous variables are presented as mean ± standard deviation. For normally distributed data, independent samples *t*-tests were used, while non-normally distributed data were analyzed using non-parametric tests. Repeated measures analysis of variance (ANOVA) was utilized to compare VAS scores, ODI scores, CRP, and CK levels at different time points within each group. Chi-square tests were employed for comparing categorical data, and non-normally distributed data were analyzed using the Mann-Whitney *U*-test. A significance level of *P* < 0.05 was considered statistically significant.

## 4 Results

A total of 115 patients were included in the final analysis, with 70 in the single-incision MIS-TLIF group and 45 in the open surgery group, six patients were lost to follow-up. Baseline characteristics including age, gender, BMI, and surgical segments showed no statistically significant differences between the two groups (*P* > 0.05), indicating comparability (Table 1). There were no statistically significant differences between the groups in terms of surgical duration, hospitalization costs, and postoperative length of stay (*P* > 0.05). The single-incision MIS-TLIF group had significantly less intraoperative blood loss and postoperative drainage compared to the open surgery group, with statistical differences observed between the groups (Table 2). Regarding complication rates (infection, thrombosis, and poor wound healing), statistical differences were observed between the two groups (Table 2). Preoperative levels of C-reactive protein (CRP) and serum creatine kinase (CK) did not differ significantly between the two groups, but postoperative increases at 1 day and 3 days were smaller in the single-incision MIS-TLIF group compared to the open surgery group, with statistical significance noted (*P* < 0.05) (Table 2). At various follow-up time points postoperatively, the single-incision MIS-TLIF group showed greater reductions in VAS scores for back pain and better improvements in ODI scores compared to the open surgery group, with statistically significant differences observed between the groups. There were no statistically significant differences between the groups in terms of VAS scores for leg pain (Table 3). According to the modified MacNab criteria, there were statistically significant differences in the excellent/good rate between the two groups, while the fusion rate showed no statistically significant difference (Table 4).

**Table 1:** Comparisons of baseline data and surgical segments.

	Single-incision MIS-TLIF group (*N* = 70)	Open surgery group (*N* = 45)	*P* > 0.05
Baseline data			
Age at surgery (year)	59.4 ± 13.54	57.6 ± 14.56	*P* > 0.05
Gender (male/female)	38(M)/32(F)	30(M)/15(F)	*P* > 0.05
BMI	27.2 ± 6.80	28.4 ± 7.23	*P* > 0.05
Number of surgical segments			
L3-4	5	2	*P* > 0.05
L4-5	36	27	*P* > 0.05
L5-S1	29	16	*P* > 0.05

**Table 2:** Comparisons of surgery-related results and laboratory results.

	Single-Incision MIS-TLIF group (*N* = 70)	Open surgery group (*N* = 45)	*P*
Surgery-related results			
Operation time (min)	115.64 ± 15.26	109.27 ± 18.42	*P* > 0.05
Intraoperative blood loss(ml)	104.5 ± 24.53	214.9 ± 32.33	*P* < 0.05
Postoperative drainage(ml)	141.4 ± 21.46	224.5 ± 25.53	*P* < 0.05
Length of hospital stay (d)	10.6 ± 1.83	11.2 ± 2.64	*P* > 0.05
Hospital costs (W)	4.3 ± 1.92	4.5 ± 2.27	*P* > 0.05
Complication	infection (0), DVT (1); poor wound healing (1)	infection (1), DVT (1); poor wound healing (2)	*P* < 0.05
Laboratory results			
CRP(preoperative) (mg/L)	4.88 ± 14.25	5.5 ± 19.5	*P* > 0.05
CRP(1d) (mg/L)	48.75 ± 45.22	94.82 ± 44.85	*P* < 0.05
CRP(3d) (mg/L)	31.14 ± 52.58	52.36 ± 54.50	*P* < 0.05
CK(preoperative) (U/L)	72.85 ± 25.59	73.21 ± 35.78	*P* > 0.05
CK(1d)	344.92 ± 79.61	457.64 ± 107.26	*P* < 0.05
CK(3d)	102.75 ± 59.40	152.75 ± 39.29	*P* < 0.05

**Table 3:** Comparisons of surgical outcomes.

Group	Preoperative	Postoperative 3 months	Postoperative 6 months	Postoperative 12 months
Back pain VAS score				
Single-Incision MIS-TLIF group	4.9 ± 0.92	2.1 ± 0.72	1.5 ± 1.03	1.4 ± 0.88
Open surgery group	4.5 ± 1.13	4.0 ± 1.55	3.4 ± 0.62	2.7 ± 0.48
*P*	*P* > 0.05	*P* < 0.05	*P* < 0.05	*P* < 0.05
Leg pain VAS score				
Single-Incision MIS-TLIF group	5.7 ± 0.83	2.2 ± 1.02	1.7 ± 0.74	1.4 ± 0.78
Open surgery group	5.5 ± 0.76	2.0 ± 0.93	1.8 ± 0.85	1.0 ± 0.26
*P*	*P* > 0.05	*P* > 0.05	*P* > 0.05	*P* > 0.05
ODI score				
Single-Incision MIS-TLIF group	68.8 ± 11.62	30.4 ± 10.13	24.5 ± 12.98	13.6 ± 13.70
Open surgery group	65.1 ± 12.23	39.2 ± 11.7	32.2 ± 13.74	20.3 ± 12.54
*P*	*P* > 0.05	*P* < 0.05	*P* < 0.05	*P* < 0.05

**Table 4:** Comparisons of 12 months after operation macNab criteria and fusion rate.

Postoperative 12 months MacNab criteria					
Group	excellent	good	fair	poor	excellent/good rate
Single-incision MIS-TLIF group	42	23	3	2	92.85%
Open surgery group	22	17	4	3	86.67%
*P*	*P* < 0.05				
Postoperative 12 months fusion rate (Modified Brantigan scoring)					
Group	1 point		2 point	3 point	4 point
Single-Incision MIS-TLIF group (*n*)	1		5	45	19
Open surgery group (*n*)	1		3	31	10
*P*	*P* > 0.05				

## 5 Discussion

With the ongoing trend of societal aging, the prevalence of degenerative lumbar spine diseases continues to rise, significantly impacting patients' daily lives and increasing societal burdens. While open surgery has demonstrated certain efficacy in treating degenerative lumbar spine diseases, it imposes considerable trauma on elderly patients, and some may find it intolerable (16). Advancements in medical technology have led minimally invasive surgery (MIS) to become an increasingly preferred choice for many patients (17). Literature reports indicate that MIS-TLIF effectively treats degenerative lumbar spinal stenosis with advantages such as minimal soft tissue damage, reduced blood loss, and faster recovery (18). This study employs a modified midline posterior approach with expandable tubular assisted by a transforaminal approach, minimizing damage to surrounding muscle tissue and enabling safe and stable access to the posterior spine for minimally invasive procedures. This approach effectively reduces patient pain, enhances surgical outcomes, and does not increase patient costs.

Based on perioperative data from two groups of patients, single-incision MIS-TLIFcan be successfully performed without extending the operative time. There was no difference in the operative time between the two groups, indicating a short learning curve for the modified single-incision MIS-TLIF. The overall surgical process is similar to open surgery, suggesting that surgeons familiar with open techniques can quickly adapt to minimally invasive procedures. Literature reports indicate that most endoscopic minimally invasive surgeries have a steep learning curve (19), but the single-incision MIS-TLIF with instrument-assisted surgery shortens this curve significantly, facilitating the clinical adoption of this technique.

Comparing intraoperative blood loss and postoperative drainage between the single-incision MIS-TLIF and open surgery groups, MIS-TLIF shows significantly less blood loss, consistent with findings from numerous clinical studies (20, 21). This reduction is attributed to the use of expandable tubular, which exert certain pressure on local muscles, thus reducing muscle bleeding during surgery. Additionally, thorough hemostasis is achieved through meticulous dissection of the muscle gap, whereas subperiosteal dissection in open surgery involves a wider exposure area, leading to higher intraoperative blood loss.

Minimally invasive surgery often signifies reduced tissue damage, yet typically incurs higher hospitalization costs (22, 23). Analyzing hospitalization expenses between two patient groups, both were comparable, despite being categorized as minimally invasive procedures, which often involve various complex instruments, potentially increasing overall hospitalization costs. However, single-incision MIS-TLIF, which utilizes equipment similar to open surgery, offers significant cost savings, which is advantageous for both individual patients and national healthcare systems.Comparing tissue damage indicators between the two groups of patients, under strictly aseptic conditions, the open surgery group exhibited significantly higher increases in CRP and CK levels at 1 day and 3 days postoperatively compared to the single-incision MIS-TLIF group. This indicates less trauma in the single-incision MIS-TLIF group, with reduced disruption to paraspinal muscles and soft tissues. Preserving paraspinal muscles during spinal surgery is crucial, as postoperative studies have shown that open posterior approaches lead to decreased multifidus muscle area post-surgery, with increased levels of fatty infiltration compared to preoperative conditions (24, 25). Literature also links increased fatty infiltration of the multifidus muscle to significant lower back pain and increased rates of lumbar spine degeneration, demonstrating a clear clinical correlation (26, 27).

Clinical efficacy indicators among both patient groups revealed significant improvements in leg pain VAS scores at 3, 6, and 12 months postoperatively compared to preoperative values, with no statistically significant differences observed between the groups at each follow-up time point. This suggests that both surgical approaches effectively decompress nerve roots with comparable outcomes. Furthermore, improvements in lumbar pain VAS and ODI scores were more pronounced in the single-incision MIS-TLIF group, showing statistically significant differences at various postoperative time points. However, there were no statistically significant differences in the interbody fusion rates at each surgical segment between the two groups at the 12-month follow-up, indicating reliable osseous fusion outcomes in both groups. Excluding efficacy differences caused by non-fusion-related factors, the potential reasons for the effectiveness of MIS-TLIF may lie in its approach through the muscle space, which protects paraspinal muscles during surgery and facilitates faster recovery with less loss of function postoperatively. Studies indicate that the multifidus muscle stabilizes the lumbar spine and may contribute to reducing the occurrence of low back pain by controlling excessive motion in the facet joints (28). Similarly, research has shown a significant association between the degree of multifidus muscle fat infiltration and postoperative low back pain and ODI scores (29, 30). Currently, there is debate over whether exercises targeting the lumbar muscles can improve multifidus muscle function (31). Some perspectives suggest that although lumbar muscle conditioning exercises may increase the size of the lumbar multifidus muscles in chronic LBP patients, this change may not correlate with clinical outcomes (32). Hence, protecting the multifidus muscle during surgery appears particularly crucial.

The study acknowledges limitations as a retrospective single-center clinical study with a limited number of cases. Future research should focus on prospective studies with larger sample sizes to validate the superiority of single-incision MIS-TLIF surgery. Long-term follow-up evaluations are also essential to assess the extended therapeutic effects. Quantifying multifidus muscle area and quality postoperatively can provide more objective and scientific evidence regarding the protective role of single-incision MIS-TLIF in muscle preservation.

## 6 Conclusion

Both surgical procedures demonstrate satisfactory clinical outcomes in alleviating pain and improving function in patients with degenerative lumbar spine disease. However, single-incision MIS-TLIF offers advantages of reduced trauma and quicker recovery, aligning more closely with current minimally invasive surgical principles. Additionally, it requires less learning time for surgeons and is associated with lower costs for patients compared to other minimally invasive surgeries, making it a foundational technique for promoting minimally invasive approaches in lumbar spine surgery.

## Data Availability

The data that support the findings of this study are not openly available due to reasons of sensitivity and are available from the corresponding author upon reasonable request.
